# The application of umbilical cord‐derived MSCs in cardiovascular diseases

**DOI:** 10.1111/jcmm.16830

**Published:** 2021-08-11

**Authors:** Yueqiu Chen, Han Shen, Yinglong Ding, You Yu, Lianbo Shao, Zhenya Shen

**Affiliations:** ^1^ Institute for Cardiovascular Science Soochow University Suzhou China; ^2^ Department of Cardiovascular Surgery of The First Affiliated Hospital Soochow University Suzhou China

**Keywords:** cardiovascular diseases, differentiation ability, immunoregulatory property, paracrine effect, umbilical cord‐derived MSCs

## Abstract

Transplantation of stem cells is a promising, emerging treatment for cardiovascular diseases in the modern era. Mesenchymal stem cells (MSCs) derived from the umbilical cord are one of the most promising cell sources because of their capacity for differentiation into cardiomyocytes, endothelial cells and vascular smooth muscle cells *in vitro*/*in vivo*. In addition, umbilical cord‐derived MSCs (UC‐MSCs) secrete many effective molecules regulating apoptosis, fibrosis and neovascularization. Another important and specific characteristic of UC‐MSCs is their low immunogenicity and immunomodulatory properties. However, the application of UC‐MSCs still faces some challenges, such as low survivability and tissue retention in a harmful disease environment. Gene engineering and pharmacological studies have been implemented to overcome these difficulties. In this review, we summarize the differentiation ability, secretion function, immunoregulatory properties and preclinical/clinical studies of UC‐MSCs, highlighting the advantages of UC‐MSCs for the treatment of cardiovascular diseases.

## 1 INTRODUCTION

Cardiovascular disease (CVD) is considered the leading cause of morbidity and mortality in humans worldwide.^1^, ^2^, ^3^ CVDs affect the structures and function of the heart or blood vessels in patients. Ischaemic heart disease (IHD), specifically myocardial infarction (MI), is the most common coronary heart disease, and the leading cause of death worldwide.^4^, ^5^, ^6^, ^7^ During CVD progression, a massive number of cardiomyocytes die and are lost, resulting in cardiac structural and functional impairment, including inflammatory cell and inflammatory pathway activation, fibroblast activation and metabolic disorders in the heart, which ultimately leads to heart failure.^8^ Necrosis of cardiomyocytes, the inflammatory response, repair, structural remodelling and hypoxia‐ischaemia status are common phenomena in heart disease.^9^, ^10^ Despite scientific progress and advancements in surgical and other insertional interventions, complete recuperation from myocardial disorders is still elusive due to the insufficiency of functional cardiomyocytes.^11^

In the field of CVDs, stem cells have received attention. Stem cell research has undergone considerable expansion since pluripotent cells were first isolated from mouse embryos in 1981.^12^, ^13^ Mesenchymal stem/stromal cells (MSCs) are derived from adult and neonatal tissues and have been intensively investigated for their use in regenerative medicine. MSCs are widely used in clinical practice because they have fewer ethical issues and safety concerns than other stem cells such as embryonic stem cells (ESCs) and induced pluripotent stem cells (iPSCs).^14^ Researchers have established many robust, practical, reproducible and low‐cost protocols for MSC isolation and storage.^15^, ^16^, ^17^, ^18^, ^19^ One kind of MSC widely used in regeneration medicine is umbilical cord‐derived MSCs (UC‐MSCs). The umbilical cord is a perinatal tissue containing 2 umbilical arteries and 1 umbilical vein, which are embedded with Wharton's jelly. MSCs can be isolated from the umbilical cord by enzymatic digestion methods.^20^ MSCs derived from UC/Wharton's jelly can differentiate into three germ layers and migrate to damaged tissue.^21^, ^22^ A study conducted by Vidal et al. demonstrated that the umbilical cord may be preferable for MSC banking purposes in research and tissue engineering compared with bone marrow.^23^ In addition, UC‐MSCs present a more primitive phenotype and exhibit longer telomeres, which enables them to achieve a greater proliferative ability and less cellular senescence.^24^

However, stem cell transplantation still faces challenges due to the low ability of some features, including the viability of cells, homing of cells to damaged locations, survival, retention in the disease environment and the paracrine effect of stem cells. Due to the issues mentioned above, there is a need for improved therapeutics based on MSCs. To date, researchers have conducted a number of animal and clinical studies to investigate the potential of UC‐MSCs for CVD treatment. In this review, we focus on the regenerative and secretive properties of UC‐MSCs. Moreover, we summarize techniques to enhance the function and clinical usage of UC‐MSCs in CVDs.

## 2 DIFFERENTIATION ABILITY OF UC‐MSCS

**FIGURE 1:**
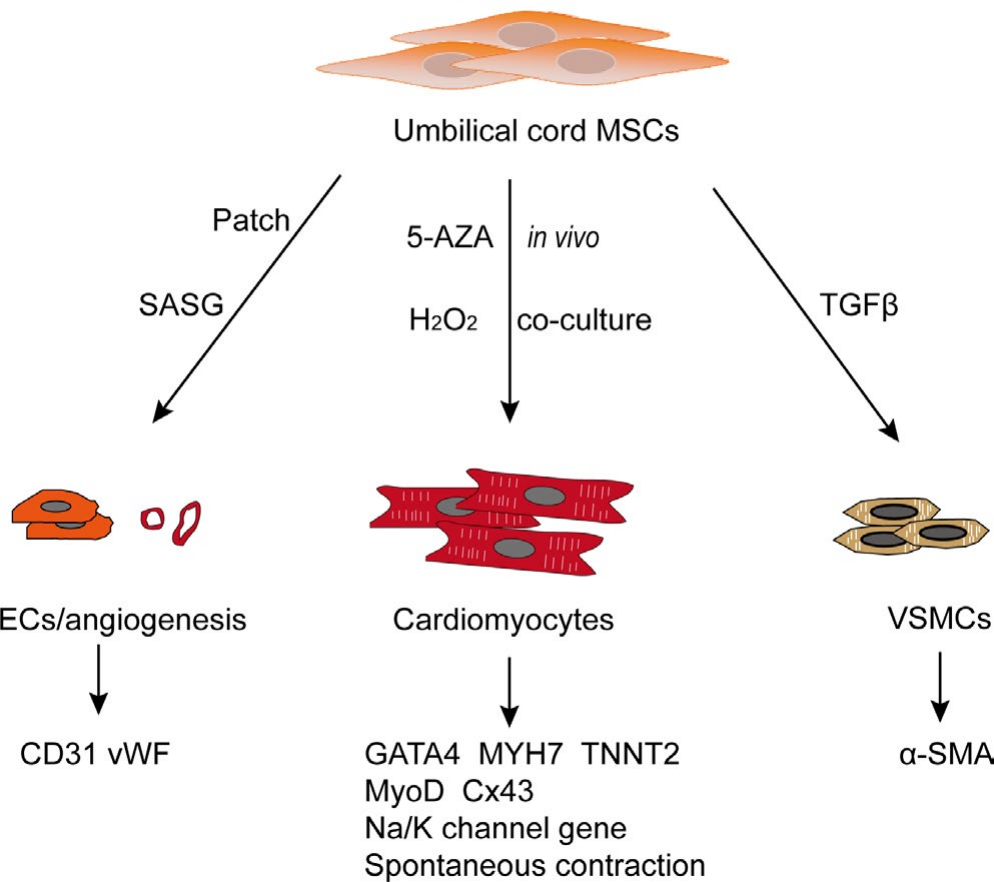
Differentiation ability of umbilical cord‐derived MSCs. UC‐MSCs can differentiate into cardiomyocytes, endothelial cells and vascular smooth muscle cells. Various reagents and materials have been used for the induction process *in vitro* and *in vivo*. Many markers have been detected to identify differentiation. 5‐AZA: 5‐azacytidine; α‐SMA: α‐smooth muscle actin; Cx43: Connexin‐43; EC: endothelial cells; GATA4: GATA‐binding protein 4; MyoD: Myogenic differentiation factor; MYH7: myosin heavy chain 7; SASG: subamnion‐cord‐lining mesenchymal stem cell angiogenic spheroids embedded within fibrin grafts; TNNT2: troponin T2; TGFβ: transforming growth factor; VSMCs: vascular smooth muscle cells; vWF: von Willebrand factor

One of the most important factors leading to CVDs is the loss of a large number of cardiomyocytes and their limited intrinsic capacity to regenerate damaged tissue. Cardiomyocytes are essential for ensuring the contraction of the chambers and efficient blood flow throughout the body.^25^, ^26^ Vascular smooth muscle cells (VSMCs) and endothelial cells (ECs) are the major cell types in blood vessels.^27^ UC‐MSCs play an important role in cardiac regenerative medicine due to their ability to differentiate into cardiomyocytes, VSMCs and ECs, which are summarized in (Figure 1).^28^, ^29^, ^30^ We also summarize the studies that induced UC‐MSCs into cardiovascular lineages in Table 1, which will be described in the following paragraphs.

**TABLE 1:** Differentiation ability of umbilical cord‐derived MSCs

Molecular	Disease	Function *in vitro*	Function *in vivo*
5‐azacytidine (5‐AZA)^33^, ^34^	Chronic HF	Enhance the expression of CM markers (NKX2.5, GATA4 and MEF2C; MYH7B and TNNT2; Cx 43)	Improve left ventricular function
H_2_O_2_ ^35^	Impaired redox environment	Enhance cardiogenic and ion‐channel gene expression (Gata4, β‐MHC, Troponin I; K and Na channel gene)	∕
*In vivo* culture^36^	Acute MI	Express CM‐specific marker CTNT	Improve myocardial perfusion and function
Foetal BM‐MSCs^39^	MI	CM muscle marker MyoD; weak positive Cx 43; spontaneous contractions cells	Improved LVEF;
Embryonic CMs & lentivirus^37^	MI	Express GATA4 and Mef2c gene; α‐MHC protein	Improve cardiac function determined as FS
cardiomyocytes^38^	∕	Spontaneous contractions CMs; enhance expression of Cx43, Mef2c, cTnT and MY6H	∕
TGFβ1^30^	Vascular grafts	SMCs display a contracting capacity	Support vascular structure formation in the matrigel plug assay *in vivo*
Hypoxic condition/ *in vivo* ^50^	MI	Form capillary‐like structure and branching points	Improve CD31+ cells; attenuate heart remodelling
UC‐MSCs^41^	Chronic myocardial ischaemia	∕	Promote collateral development and myocardial perfusion; improve systolic thickening fraction; enhance the expression of vWF
UC‐MSC patch^43^	MI	∕	Improve cardiac contractile function; enhance neovessels
SASG^44^	HF	∕	Preserve cardiac function and induce myocardial revascularization; attenuate cardiac fibrosis

Abbreviations: CM: cardiomyocytes; Cx43: Connexin‐43; GATA4: GATA‐binding protein 4; HF: heart failure; LVEF: left ventricular eject fraction; MEF2C: myocyte enhancer factor 2C; MHC: Myosin heavy chain; MYH7B: myosin heavy chain 7B; MyoD: Myogenic differentiation factor; NKX2.5: NK2 homeobox 5; SASG: subamnion‐cord‐lining mesenchymal stem cells angiogenic spheroids embedded within fibrin grafts; vWF: von Willebrand factor; α‐SMA: α‐smooth muscle actin.

## 3 INDUCTION OF UC‐MSCS INTO CARDIOMYOCYTES

MSCs, as an alternative cell source for use in clinic, have been investigated for use in regenerative medicine since their initial description in 1995.^31^ The safety and efficacy of MSCs have been shown in several studies on cardiac regeneration.^28^, ^29^ In the cardiomyocyte induction process, some markers have been identified to indicate the differentiation stage: initiation or maturity. NKX2.5, myocyte enhancer factor 2C (Mef2c) and GATA4 as early differentiation markers indicate the initiation of cardiomyocyte differentiation. Cardiac troponin T (cTnT), heavy chain cardiac myosin (MYH6) and connexin‐43 (Cx43), mature cardiomyocyte markers, are used to indicate maturity.^32^

A series of studies have been conducted to regenerate cardiomyocyte‐like cells from UC‐MSCs *in vitro* and *in vivo*. Treatment with 5‐azacytidine (5‐AZA) induced UC‐MSCs to differentiate into cardiomyocytes, demonstrated by the expression of the myogenesis transcription factors, such as NKX2.5, GATA4 and Mef2c, and other specific markers, such as TNNT2, MYH7B and Cx43. UC‐MSCs can differentiate into cardiomyocytes and improve left ventricular function and quality of life in patients with chronic stable heart failure after cell transplantation.^33^, ^34^ Another study conducted by Subramani et al. showed that an impaired redox environment induced by H_2_O_2_ significantly increases the expression of the cardiac genes‐GATA4, M1c2a, β‐MHC and Troponin I in UC‐MSCs. Ion channels in cardiomyocytes are indispensable in cardiac regenerative medicine. Notably, the gene expression of all subunits of the K+ channel and one Na channel was improved by H_2_O_2_ in UC‐MSCs compared with the basal control.^11^, ^35^ In addition to the induction of UC‐MSCs into cardiomyocytes *in vitro*, Zhang et al. discovered that CM‐Dil‐labelled UC‐MSCs expressed the cardiomyocyte‐specific marker cTNT in mini swine acute MI model six weeks after cell transplantation.^36^

Beyond the reagents used to induce the differentiation of UC‐MSCs, a coculture system with cardiomyocytes or bone marrow MSCs (BMSCs) has produced exciting results. Yannarelli et al. reported that when UC‐MSCs were cocultured with rat embryonic cardiomyocytes, the expression levels of the cardiomyocyte transcription factors GATA4 and Mef2c in UC‐MSCs were increased 7.6‐ and 3.5‐fold at 7 days, respectively. A higher frequency of α‐MHC protein was detected in UC‐MSCs than in BMSCs.^37^ Moreover, the cardiac regeneration effect was detected with cell transplantation *in vivo* by intramyocardial injection after AMI. UC‐MSCs exhibited a greater improvement in cardiac function than BMSCs, determined as fractional shortening (FS).^37^ Unfortunately, they induced partially mature cardiomyocytes. Spontaneously contracting cardiomyocytes from human MSCs were not reported until 2017 by Szaraz et al.^38^ Szaraz and colleagues cocultured UC‐MSCs with rat cardiomyocytes, which were used as a feeder layer, and they induced s spontaneous contraction of cardiomyocytes from human UC‐MSCs. They found higher expression of the Cx43 gene and Mef2c protein in UC‐MSCs than BMSCs. Cardiomyocyte‐specific regulatory and structural protein genes such as cTnT and MY6H were also upregulated in the coculture system. Inspiringly, the authors observed spontaneous contraction within 1 week.^38^ López et al conducted a similar coculture experiment to investigate the long‐term therapeutic effect of UC‐MSCs following MI. They demonstrated that UC‐MSCs labelled with SP‐Dil migrated to the peri‐infarction region four days after IV injection. Furthermore, they found an improvement in the ejection fraction 25 to 31 weeks after UC‐MSC treatment *in vivo*. UC‐MSCs cocultured with fatal BMSCs formed myotube structures in two to three days and spontaneous contractions were observed in five to seven days with MyoD‐positive muscle markers *in vitro*.^39^ The above results reinforce the concept that UC‐MSCs are a novel and promising source of autologous cells for cardiac tissue engineering.

## 4 INDUCTION OF UC‐MSCS INTO VSMCS

VSMCs are another important component in the cardiovascular system and are critical for the maintenance of physiological functions of the blood vessel wall. Moreover, tissue‐engineered vascular grafts with long‐term patency are greatly needed in clinical settings, and smooth muscle cells (SMCs) are a critical graft component. Gu et al. found that human UC‐MSCs abundantly express the SMC markers α‐smooth muscle actin (αSMA), smooth muscle protein 22 (SM22), calponin and smooth muscle myosin heavy chain (SMMHC) at both the gene and protein levels in response to stimulation with transforming growth factor‐β1 (TGFβ1).^30^ Functionally, MSC‐derived SMCs displayed contracting capacity *in vitro* and supported vascular structure formation in the Matrigel plug assay *in vivo*. Mechanistically, they demonstrated that miR‐503 promoted SMC differentiation by directly targeting SMAD7 and that miR‐503 expression was SMAD4‐dependent.^30^

## 5 INDUCTION OF UC‐MSCS INTO ECS

Endothelial cells form a single‐cell layer that is a selective blood‐tissue barrier regulating exchanges between the bloodstream and the surrounding tissues. Physiologically, ECs take part in angiogenesis, vasculogenesis and vasoregulation in the cardiovascular system. Researchers have found that UC‐MSCs can enhance the abilities of endothelial cells. Conditioned medium produced by UC‐MSCs enhances the formation of capillary‐like structures and branching points by endothelial cells *in vitro*. Furthermore, conditioned medium collected under hypoxic conditions shows an increased number of capillary‐like structures compared with normoxic conditions. UC‐MSC transplantation improved CD31^+^ cells, especially in the myocardium adjacent to the ligation region in an MI model.^40^ A similar result was found by Liu et al. with von Willebrand factor (vWFs) expression and collateral development in chronic myocardial ischaemia.

Furthermore, vWFs were found in the border zone of the infarction zone with CM‐Dil positivity, which suggested the differentiation of UC‐MSCs into endotheliocytes.^41^ To enhance the survival and retention of transplanted cells, 3D culture and cell patches have been developed.^42^, ^43^, ^44^ Li et al. transplanted UC‐MSCs patches into an MI model, and they observed CD31^+^ blood vessel‐like structures and blood cells inside.^43^ The SASG patch used by Martinez et al. enhanced the vascular density presented by RECA‐1^+^ in the left scar area. Moreover, a large number of Dil+functional blood vessels with seemingly active angiogenic sprouting were observed after transplantation of UC‐MSCs with Dil labelling.^44^

## 6 PARACRINE EFFECTS OF UC‐MSCS

**FIGURE 2:**
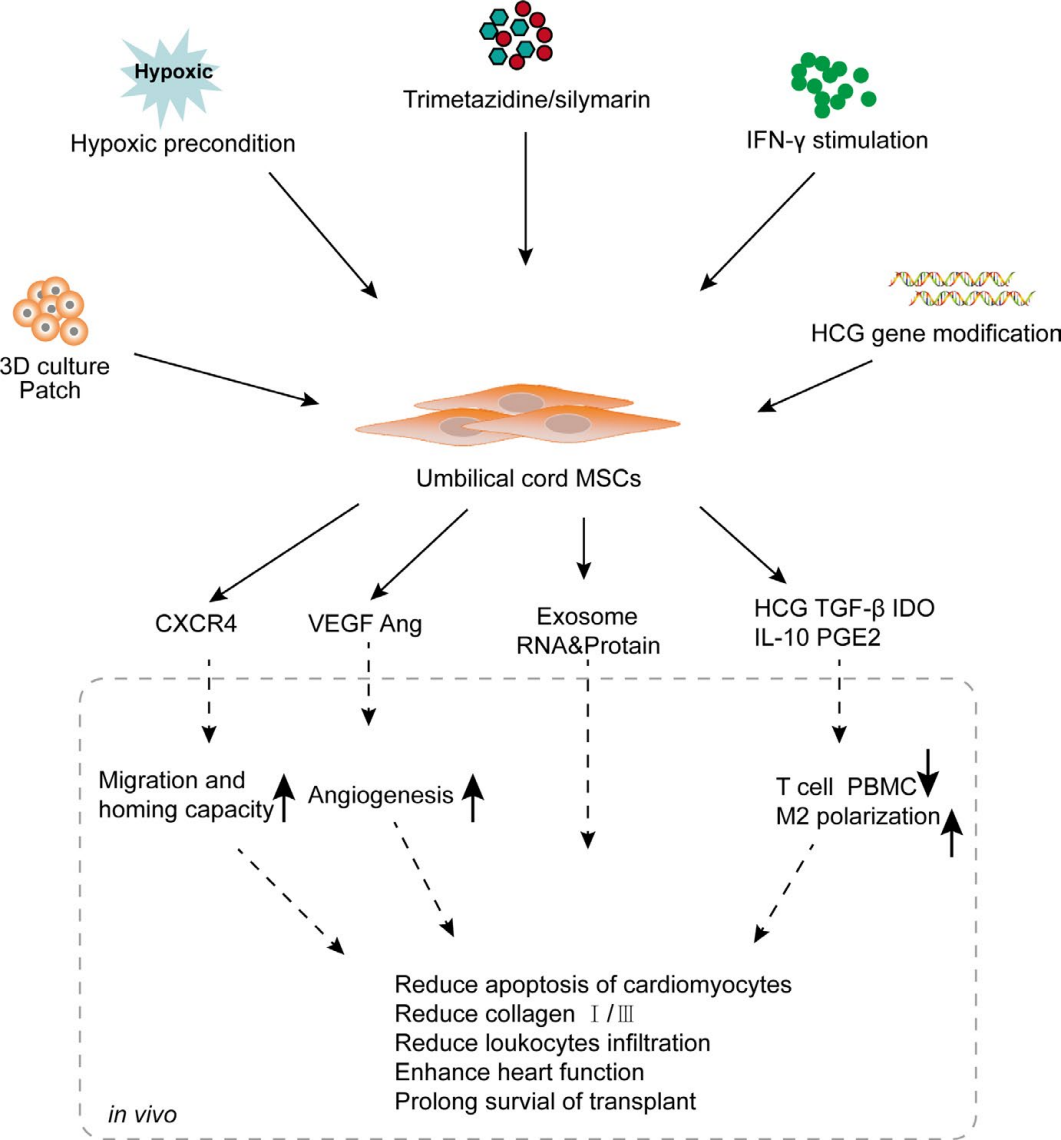
Pretreatment, paracrine and therapeutic effects of UC‐MSCs in cardiovascular diseases. Various reagents and culture conditions were used to enhance the paracrine effect of UC‐MSCs. These paracrine factors enhanced the migration and homing capacity, angiogenesis effect and immunoregulatory ability of UC‐MSCs. Ang: angiotensin; CXCR4: Cxc chemokine receptor 4; HCG: hepatocyte growth factor; IDO: indoleamine 2,3‐dioxygenase; M2: macrophage subtype Ⅱ. PGE2: prostaglandin E2; PBMC: peripheral blood mononuclear cell; VEGF: vascular endothelial growth factor

Although UC‐MSCs differentiate into cardiovascular‐related cells, their ability and specialized target cell numbers are limited. It is widely accepted that the majority of UC‐MSC‐derived myocardial recovery *in vivo* depends on paracrine effect.^40^, ^41^ UC‐MSCs showed higher TGF‐β3 and hepatocyte growth factor (HGF) gene expression than BMSCs, which greatly influences the paracrine effects of UC‐MSCs.^33^ The paracrine effects of UC‐MSCs, including neovascularization and antiapoptosis effects, anti‐fibrosis effects and immunoregulatory effects, are summarized in **(**Figure 2 **)** and will be described in the following paragraphs (Table 2).

**TABLE 2:** Paracrine effects of UC‐MSCs

Molecular	Disease	Signalling pathways	Function *in vitro*	Function *in vivo*
HGF, TGF‐β3, IDO, PGE2^33^	Human heart failure	∕	∕	Improve left ventricular function and quality of life
VEGF and Ang^41^	Chronic MI	∕	∕	Promote angiogenesis, reduced apoptosis and fibrosis
Condition medium^46^	Irradiation myocardial fibrosis model	NF‐κB	Reduce Col1A1 and Col1A2 deposition; reduce TGF‐β1, IL−6 and IL−8	∕
UC‐MSCs^48^	DCM	TGF‐β/ERK	∕	Reduce collagen I/III, TGF‐β1, TNF‐α and connective tissue growth factor; restore cardiac function
UC‐MSCs/ IFNγ stimulation^50^	Xenogeneic transplantation	∕	Release high level of TGF‐β, IL−10 and IDO	UC‐MSCs were rejected more slowly
CL‐MSCs^51^	Mixed lymphocyte reaction	∕	Reduce the release of IFNγ by lymphocytes	Prolong survival time in xenogeneic BALB/c mice
Condition medium^54^	Cardiac transplantation	∕	Inhibit the proliferation of PBMC; reduce the production of IL−2 and IFNγ by PBMC	Prolong survival time of cardiac allograft; reduce IL−2 and IFNγ, enhance IL−10 and TGFβ1 in cardiac allograft
UC‐MSCs^56^	MI	∕	∕	Reduce leukocyte infiltration and CD11b^+^ cells; increase M2 macrophages; improve heart function

Abbreviations: Ang: angiotensin; CL‐MSCs: umbilical cord lining MSCs; Col1A1: collagen type I alpha 1 chain; DCM: dilated cardiomyopathy; ERK: signal‐regulated kinase; HCG: hepatocyte growth factor; IDO: indoleamine 2,3‐dioxygenase; M2: type Ⅱ macrophage; MI: myocardial infarction; NF‐Κb: nuclear factor‐κB; PBMC: peripheral blood mononuclear cell; PGE2: prostaglandin E2; TGFβ: transforming growth factor; UC‐MSC: umbilical cord‐derived MSC; VEGF: vascular endothelial growth factor.

## 7 NEOVASCULARIZATION AND ANTIAPOPTOSIS EFFECTS

Hypoxia‐ischaemia and apoptosis of cardiomyocytes are common situations that occur in CVDs. Neovascularization is the process by which new vascular structures assemble that support blood and oxygen for the heart. Vasculogenesis occurring exclusively during embryogenesis and angiogenesis in adults is now recognized as two primary mechanisms that contribute to neovascularization in a single microenvironment.^45^ During the process of CVD development, the magnitude of cardiomyocyte loss and fibrotic scar formation result in diminished heart pumping capacity and ultimately lead to heart failure. Thus, it is essential to increase angiogenesis and reduce the apoptosis of cardiomyocytes.

Diana et al. found that UC‐MSCs have a proangiogenic, antiapoptotic function and attenuate remodelling after myocardial infarction. First, they transplanted UC‐MSCs into the myocardium via intramyocardial injection and discovered that CD31 expression in the infarcted and peri‐infarcted regions was enhanced. In addition, the number of apoptotic cells in the apex of UC‐MSC‐transplanted hearts was reduced by 39.0% *in vivo*.^40^ The effect of UC‐MSCs was also detected in a porcine model induced by an android constrictor, and the authors demonstrated that UC‐MSC injection through the left main coronary artery could promote angiogenesis with high VEGF and Ang expression.^41^

Because of the specificity of the heart, biomaterials were used to enhance the therapeutic effect of UC‐MSCs. 3D cardiac tissue engineering patches combined with stem cells and biomaterials first became a promising technology decades ago and significantly enhance the retention of stem cells. A study presented by Li et al. used biocompatible biomaterials pretreated with UC‐MSCs to ameliorate the inflammation and rejection of foreign material.^43^ The patch integrated into the local heart tissue and constructed neovessels, which were positive for CD31 *in vivo*. Moreover, the left ventricular wall and LVEF were improved in the UC‐MSC patch group.^43^ Similarly, Martinez et al. embedded fibrin grafts (SASGs) to facilitate donor cell survival in a rat heart failure model.^44^ First, SASG was constructed of UC‐MSCs and human umbilical vein ECs (HUVECs), and then, cells were embedded in a fibrin matrix. SASG preserved cardiac function by increasing fractional shortening, inducing myocardial revascularization presented by α‐SMA^+^ and GFP^+^ blood vessels, and promoting cardiac fibrosis. In addition, the RECA‐1^+^ blood vessels were found in the LV scar area and graft area implying the fusion of recipient and donor cells.^44^

## 8 ANTIFIBROSIS EFFECTS

Fibrosis is a compensatory consequence in CVDs, which affects cardiac contractility and develops into heart failure. Liu et al. transplanted UC‐MSCs to treat chronic myocardial ischaemia. They demonstrated that transplantation of UC‐MSCs reduced fibrosis and apoptosis *in vivo*.^41^ Irradiation‐induced myocardial fibrosis (IMF) with human cardiac fibroblasts was ameliorated with conditioned medium secreted from UC‐MSCs by inhibiting the NF‐κB signalling pathway.^46^ In the study, the author demonstrated that the medium reduced Col1A1 and Col1A2 deposition and reduced profibrotic TGF‐β1, IL‐6 and IL‐8 levels, which are crucial in promoting the proliferation of fibroblasts and driving differentiation of fibroblasts into myofibroblasts. Furthermore, they found that the medium prevented the activation of the NF‐κB signalling pathway and its proinflammatory actions induced by irradiation by reducing the level of NF‐κB p65.^46^ MicroRNAs derived from UC‐MSCs, such as miR‐21, miR‐23a, miR‐125b and miR‐145, were demonstrated to suppress myofibroblast formation by inhibiting excess α‐SMA and collagen deposition, which was associated with the activity of the TGF‐β2/SMAD2 pathway *in vitro* and *in vivo*. This is the first time that microRNAs derived from UC‐MSCs were examined by high‐throughput sequencing, with a group of specific microRNAs.^47^ Moreover, UC‐MSCs can also reduce fibrosis in a rat dilated cardiomyopathy model induced by myosin injections. In a study, UC‐MSCs reduced tumour necrosis factor‐α (TNF‐α), transforming growth factor‐β1 (TGF‐β1) and collagen I/III expression at the gene and protein levels via the TGF‐β1/ERK1/2 signalling pathways.^48^

## 9 IMMUNOREGULATORY EFFECTS

UC‐MSCs exhibit relatively low immunogenicity as a result of their limited expression of MHC I, lack of HLA‐A, MHC II molecules and costimulatory antigens, and immunosuppressive secretome and HLA‐G.^33^, ^48^ Moreover, UC‐MSCs maintain immunomodulatory molecules (HLA‐G, HLA‐E and HLA‐F) but not HLA‐DR (MHC class Ⅱ) even after differentiation *in vitro*.^49^

Deus et al. systematically compared the tolerogenic properties, immunogenicity and immunosuppressive properties of UC‐MSCs and BM‐MSCs derived from patients >65 years of age.^50^ The results showed that UC‐MSCs have higher tolerogenic properties, as indicated by the release of the tolerogenic cytokines TGF‐β and IL‐10, which were significantly enhanced by IFN‐γ treatment.^50^ UC‐MSCs were rejected more slowly than BM‐MSCs in immunocompetent BALB/c mice. Moreover, the proliferation of PBMCs was inhibited, and the inhibitory effect was enhanced under IFN‐γ treatment.^50^ MSCs derived from different parts of gestational tissue in humans have different immunogenicity and immunomodulatory capabilities. Stubbendorff M et al. demonstrated that MSCs derived from umbilical cord lining (CL‐MSCs) have a higher ability to dampen Th1 and Th2 responses in xenogeneic BALB/c mice, mostly dependent on HLA expression.^51^

In addition to the source of MSCs, the culture medium influences the paracrine effect of UC‐MSCs. Extracellular vehicles (Evs) derived from UC‐MSCs cultured under diverse conditions have different effects, such as cardiomyogenic and angiogenic potential, and immunogenic capacity. Thus, the conscious selection of cell culture conditions is required for clinical practice. Bobis‐Wozowicz et al. reported that UC‐MSCs‐secreted Evs have the highest RNA content in MSCs cultured in GM‐CD™ Bullet Kit and StemXVivo™ media. Furthermore, they showed that the secretion of anti‐inflammatory cytokines is most pronounced when MSCs are cultured in StemPro® MSC SFM media.^52^ Evs derived from MSCs cultured in PPRF‐msc6‐composition and StemPro^®^ MSC SFM media have the highest expression levels of GATA4 and NKX2.5, showing cardiomyocyte differentiation potential.^52^

A study conducted by Fazzina et al. demonstrated that UC‐MSCs showed the most prominent immunosuppressive effect *in vitro* compared with BMSCs and AT‐MSCs with the greatest proportion of immune‐related markers CD200, CD273 and CD274. Moreover, UC‐MSCs inhibited lymphocyte proliferation in the coculture system *in vitro*.^53^ MHC class II molecules, which present antigens to CD4 positive lymphocytes, are critical for the generation and maintenance of immune responses. In a cardiac transplantation model with MHC class II overexpression in the vascular endothelial cells of the donor, Qiu et al. demonstrated that repeated infusion of UC‐MSCs reduced MHC class II expression on vascular endothelial cells and prolonged the survival time of cardiac allografts *in vivo*. Furthermore, more in‐depth research suggested that regulatory cytokines such as IL‐10 and TGF‐β1 were increased and that proinflammatory cytokines such as IL‐2 and IFNγ were decreased in cardiac allografts.^54^, ^55^ The proliferation of PBMCs cultured in the conditioned medium of UC‐MSCs was inhibited, and the production of IL‐2 and IFNγ was suppressed *in vitro*. These results suggested that the factors derived from MSCs may play an important role in the function of MSCs.^54^ Another study conducted by Dayan et al. demonstrated that UC‐MSCs reduced leukocyte infiltration and the total number of CD11b^+^ cells in infarcted myocardial tissue; however, F4/80^+^CD206^+^ macrophage infiltration in infarcted tissue was significantly higher. Furthermore, UC‐MSCs could improve short‐term cardiac function presented by LV fractional shortening (FS), decrease long‐term cardiac remodelling presented by decreased septum thickness and reduce the apoptosis of cardiomyocytes presented by Bax/Bcl2.^56^ Other studies have shown that MSCs constitutively express COX1, COX2, TGF‐β and IL‐10 and release a large amount of PGE2 to inhibit the proliferation of splenocytes.^57^, ^58^ From the results above, it is speculated that the mechanisms used by UC‐MSCs seem to be paracrine effects.

## 10 CULTURE AND MODIFICATION OF UC‐MSCS

It has been demonstrated that MSCs derived from perinatal tissues have a high proliferative, antiapoptosis and anti‐senescence function, making them more suitable for use in the clinic.^59^ To enhance the survival, paracrine and therapeutic effects of UC‐MSCs, a series of studies was conducted, as summarized in Table 3 and presented in the following paragraphs (Figure 2).

**TABLE 3:** Cultivation and modification of UC‐MSCs

Molecular	Disease	Signalling pathways	Function *in vitro*/*in vivo*
3D culture^42^	∕	TLR signalling pathway	*In vitro*: Enhance expression of CD34 CD271, TLR and CXCR4 *In vivo*: Promote their migratory and homing capacity in BALB/c mice
Hypoxic precondition^65^	H9C2 in hypoxic condition	PI3K/Akt/mTOR pathway	*In vitro*: Increasing exosomes secretion of UC‐MSCs
Silymarin^66^	UC‐MSC with serum deprivation	∕	*In vitro*: Promote the proliferation of human UC‐MSCs; inhibit the serum deprivation‐induced apoptosis of MSCs; enhance the expression of BAX
Trimetazidine^67^	MI	AKT pathway	*In vitro*: Rescue the apoptosis of MSCs; increase the paracrine functions: increase expression of LIF, TGFβ, VEGF, IGF−1, CCR7, CCR8; decrease expression of TLR4, MICA, IL−23.
IFN‐γ stimulation^50^	∕	∕	*In vitro*: Enhance the immunosuppressive phenotype of MSCs; downregulate HLA‐DR expression; increase IDO production
HGF^68^	MI	∕	*In vitro*: Increase HGF, EGF, bFGF and VEGF; reduce the level of p‐Akt and the ratio of Bax/Bcl2 *In vivo*: Improve heart function; reduce cardiomyocyte apoptosis; enhance angiogenesis

Abbreviations: AKT: serine/threonine kinase; BAX: BCL2‐associated X; BCL2: apoptosis regulator; CCR: C‐C motif chemokine receptor; CXCR4: chemokine (C‐X‐C motif) receptor; For other abbreviations, see Tables 1 and 2; HGF: hepatocyte growth factor; LIF: leukaemia inhibitory factor; MICA: MHC class I polypeptide‐related sequence A; PI3K: phosphatidylinositol 3‐kinase; TLR: Toll‐like receptor.

## 11 IMPROVEMENT OF UC‐MSC STATUS

Cell viability and proliferation are important for the usage of UC‐MSCs, especially for cells used in the clinic. Zhou et al. showed that UC‐MSCs cultured in a 3D system, simulating *in vivo* conditions, decreased the expression of CD105 and enhanced the expression of CD34 and CD271. Moreover, both the mRNA and protein expression levels of TLR and CXCR4, two important receptors regulating the migration ability of MSCs, were significantly increased in the 3D‐cultured UC‐MSCs. The results were strengthened by the enhanced migratory and homing capacity observed *in vivo*, possibly mediated by CXCR4.^42^

Moreover, the adhesive and migratory properties of MSCs may influence their fate and efficacy when injected into a disease environment. Alanazi and colleagues recently found that UC‐MSCs showed the most efficient capture from flow and less spreading but more rapid migration ability compared with BMSCs, which might be associated with more effective delivery from the circulation into damaged tissue.^60^ They further investigated whether cell size affected the level of adhesion by measuring the diameter of adherent cells from the images taken immediately at the end of a perfused bolus. The average diameters were less than those of the original samples, and the cell diameter of UC‐MSCs was approximately 21.2 µm at the original sample and 17.8 µm when adherent to collagen, which was smaller than that of BMSCs.^60^ Cell behaviour differs between *in vivo* and *in vitro* cultures with a conventional two‐dimensional culture system in terms of phenotypic marker expression, homing and migratory capacity.^61^, ^62^

To optimize the most suitable transport and storage conditions for the usage of MSCs, Celikkan et al. designed a study to investigate four transport/storage medium compositions, two temperatures and storage intervals of up to 36 h. They demonstrated that Ringer's lactate (1% human serum albumin) medium at 2–10℃ is most suitable for cell viability, proliferation capability, karyotype stability and surface marker expression.^63^ Furthermore, it was meaningful to use these conditions in a clinical trial (ClinicalTrials.gov Identifier NCT02323477).^64^

## 12 MODIFICATION OF UC‐MSCS

Hypoxic preconditioning, pharmacological treatment and engineered gene modification have been performed to enhance the antiapoptosis/fibrosis and paracrine effects of UC‐MSCs. Liu et al. reported that hypoxic preconditioning increased the exosome secretion of UC‐MSCs, strengthened antiapoptosis effect by downregulating the expression of cleaved caspase 3 and regulated autophagy by downregulating the ratio of LC3B‐II/I and the expression of p62 in H9C2 cells via the PI3K/Akt/mTOR pathway.^65^ The pharmacological treatment of UC‐MSCs with silymarin and trimetazidine has increased the survival and paracrine function of implanted cells.^66^, ^67^ Silymarin has an antiapoptosis effect in UC‐MSCs under serum deprivation conditions by elevating the expression of BAX.^66^ Trimetazidine protected MSCs against apoptosis by increasing Bcl2 and p‐AKT. Moreover, the paracrine effects were greatly increased including cell cytokines, chemokines, growth, inflammation and apoptosis factors.^67^ Apart from pharmacological treatment, pretreatment with inflammatory factors, such as IFN‐γ, has increased the immunogenicity and tolerance of UC‐MSCs by upregulating the expression of HLA class Ⅰ, intracellular HLA‐G and surface HLA‐E. At the same time, IFN‐γ can enhance the levels of IL‐10, TGF‐β and IDO, which are important immunosuppressor factors for MSCs.^50^ To enhance the survival of MSCs post‐transplantation, Zhao et al. overexpressed hepatocyte growth factor (HGF) in UC‐MSCs and transplanted these MSCs into the pre‐infarct region in mice following MI. High HCG expression in MSCs improved heart function with reduced cardiomyocyte apoptosis and enhanced angiogenesis *in vivo*.^68^ Conditioned medium from UC‐MSCs with high HCG expression contains higher levels of EGF, bFGF and VEGF and lower levels of p‐Akt and a lower ratio of Bax/Bcl2.^68^

## 13 CLINICAL TRIALS WITH UC‐MSCS

Credible evidence from transplantation studies with UC‐MSCs in cardiovascular diseases has propelled several clinical applications to investigate their safety and for in allogeneic therapeutic purposes due to their immune‐privileged status.^14^, ^69^, ^70^, ^71^ UC‐MSCs can be administered via minimally invasive procedures such as coronary artery and direct myocardium injection with minimal complications. Thus far, no clinical cases of UC‐MSC malignant transformation have been reported. Various completed and ongoing clinical trials have been conducted to investigate the safety and efficacy of UC‐MSCs in patients with CVDs, which are summarized in Table 4.^71^, ^72^, ^73^, ^74^

**TABLE 4:** Completed and ongoing trials of UC‐MSCs in heart disease

Clinicaltrials.gov identifier	Disease type	Study design	Route of delivery	End‐point	Enrolled number	Status
NCT01739777^33^	Chronic stable heart failure	Randomized controlled trial; phase Ⅰ/Ⅱ;	Intracoronary injection	Safety; NYHA; LVEF	22	Completed
NCT01291329^75^	ST‐elevation MI	Randomized; parallel assigned; phase Ⅱ	Intracoronary injection	Safety; LVEF; PET; SPET	160	Completed
NCT03902067	Acute MI	Randomized; parallel assigned; phase Ⅰ	Injection into blood vessels through an administration catheter	Safety; LVEF	40	Not yet recruiting
NCT02323477^64^	Chronic ischaemic cardiomyopathy	Randomized; parallel assigned; single; phase Ⅰ/Ⅱ	Intramyocardial injection	LVEF; LVED; LVES	79	Unknown
NCT03533153	MI	Randomized; parallel assigned; phase Ⅰ/Ⅱ	Intravenous injection	Safety; MACE; heart function; infarct size; 6‐min walk test; serum BNP	90	Not yet recruiting
NCT04011059	Heart failure of coronary artery disease	Randomized; parallel assignment; phase Ⅰ/Ⅱ;	Myocardium injection	LVEF; 6‐min walk test; mortality at 3 and 12 months	40	Not yet recruiting
NCT03180450	Heart Failure	Randomized; parallel assignment; phase Ⅰ/Ⅱ;	Intravenous transplantation	Heart colour ultrasound evaluation; single therapy effectiveness evaluation	60	Unknown
NCT02635464	Chronic ischaemic cardiomyopathy	Randomized; parallel assignment; phase Ⅰ/Ⅱ;	Inject in the infarct region	LVEF; infarct size; NYHA; CCS	45	Unknown
NCT02439541	Chronic ischaemic heart disease	Randomized; parallel assignment; phase Ⅰ/Ⅱ;	Intracoronary infusion	MACE; six‐minute walk test; SPECT; echocardiography; NYHA	40	Unknown

Abbreviations: BNP: B‐type natriuretic peptide; CCS: Canadian Cardiovascular Society Angina Grading Scale; LVED: left ventricular end‐diastolic diameter; LVEF: left ventricular ejection fraction; LVES: left ventricular systolic diameter. MI: myocardial infarction; MACE: major adverse coronary events; NYHA: New York Heart Association functional class; PET: positron emission computed tomography; SPECT: single photon emission computed tomography.

A study conducted by Musialek et al. demonstrated the feasibility and procedural safety of UC‐MSCs, and no clinical symptoms or significant arrhythmias occurred. This was the first‐in‐man trial in an acute MI therapy with UC‐MSCs.^74^ A randomized and controlled clinical study with a sizable sample was conducted in 116 acute ST‐elevation MI patients by Gao et al (NCT01291329). Adverse events including death, recurrence of AMI and rehospitalization due to heart failure, and laboratory tests including tumour, immune and haematologic indexes were not different between the UC‐MSC group and placebo group. The LVEF increased significantly while LV end‐systolic volumes and LV end‐diastolic volumes decreased significantly in the UC‐MSC group after 18 months of cell therapy.^75^

The safety and efficacy of UC‐MSCs were also tested by Bartolucci et al. through intravenous infusion in patients with chronic stable HF (NCT01739777).^33^ First, they found no acute adverse events associated with the infusion of allogenic UC‐MSCs. Moreover, they found an improvement in LVEF and LVEDV and quality of life in the UC‐MSC group. Another study conducted in chronic systolic HF by Zhao et al. evaluated the effectiveness of UC‐MSCs through intracoronary transplantation. Among the 30 recipients, 29 patients had no adverse reactions, heart palpitations, chest pain, chest tightness, dyspnoea or other symptoms, while one experienced chest discomfort and showed ST changes. The cardiac structure shown by LVEDD 6 months after transplantation was enhanced, as were the LVEF and 6‐min walking distance 1 month after transplantation. In addition, the NT‐proBNP level, mortality and readmission were decreased 6 months after transplantation, even though the difference was not statistically significant.^76^ Another controlled and randomized trial in combination with coronary artery bypass grafting was conducted with chronic ischaemic cardiomyopathy through intramyocardial delivery by Can et al. in 79 patients (NCT02323477). The authors found no significant difference in the adverse cardiac event rate defined as the composite incidence of death, hospitalization for worsening heart failure, nonfatal recurrent MI and ventricular arrhythmia. There was a significant elevation in LVEF and significant increases in regional contractility within the infarcted area.^64^ Although no direct MSC‐related adverse effects have been reported, low survival and retention restrict the systematic application of UC‐MSCs. Moreover, long‐term follow‐up for patients receiving MSCs is needed including infusion reaction, pulmonary embolisms and ectopic tumour formation.

## 14 PROBLEMS AND PROSPECTS

The major mechanism of MSC transplantation for myocardial repair is the paracrine effect, and the immunomodulation effect is one of the most important specific characteristics of UC‐MSCs, which is more significant than that of other tissue‐derived MSCs. Many reports have shown that paracrine factors such as miRNAs, lncRNAs, circRNAs, exosomes and cytokines can transmit the effect of UC‐MSCs.^52^ In addition, mitochondria are important organelle regulating metabolism and have been reported to be included in extracellular vesicles of MSCs, especially iPSC‐derived MSCs. Moreover, mitochondrial donation of MSCs derived from iPSCs has been used to treat anthracycline‐induced cardiomyopathy, retinal ganglion cell degeneration and cigarette smoke‐induced lung damage.^77^, ^78^, ^79^ In addition to iPSC‐MSCs, UC‐MSCs can facilitate renal repair in rats by regaining mitochondrial mass and the function of damaged tubular cells through paracrine effects.^80^

Many signalling pathways take part in the regulation and mediate the function of UC‐MSCs. As mentioned above, the TGF‐β/ERK and AKT signalling pathways influence the paracrine action and function of UC‐MSCs.^48^, ^67^ Zhang et al. reported that the Rap1‐NF‐κB signalling pathway regulated the paracrine activity of bone marrow MSCs and had a therapeutic effect in myocardial infarction.^81^ Moreover, Rap1 deficiency causes defects in paracrine function and impairs the immunoregulatory potency of bone marrow MSCs.^82^ However, Rap1 has not been studied in UC‐MSCs, and the effects of the Rap1 and Rap1‐NF‐κB signalling pathways may show great potential for UC‐MSCs in the therapy of CVDs.

MSCs have been studied in many preclinical studies and have shown some good performance with no safety issue. However, quality control of UC‐MSCs in clinical studies is more difficult, and UC‐MSCs become senile from batch to batch. Some research groups modified MSCs with ERBB4 to enhance the activity and angiogenesis of aged MSCs,^83^ which brings potential safety risks. Moreover, an extensive number of MSCs is a prerequisite for cell therapy. The primary generation of MSCs derived from tissues is limited and the passaged cells may become senile. To overcome these limitations, iPSC‐derived MSCs beginning at the single‐cell level have been proposed. These iPSC‐MSCs have a higher proliferation potential than bone marrow MSCs and regulate the polarization of RAW264.7 cells.^84^, ^85^ The safety and efficacy of iPSC‐MSCs have been demonstrated in clinical trials of graft versus host disease.^86^ However, the effect of the regulatory pathway has not been compared between UC‐MSCs and iPSC‐MSCs, including in CVDs.

Overall, UC‐MSCs have the advantages of rich sources, easy isolation, low immunogenic properties and potent immunomodulatory effects. Moreover, transplanted UC‐MSCs can home to infarcted myocardial tissue and contribute to the repair of infarcted myocardium. The loss of cardiomyocytes is a major reason for inflammation and heart remodelling in CVDs. However, due to the limited differentiation ability of MSCs into cardiomyocytes, much more work needs to be done, such as enhancing the antiapoptosis/anti‐fibrosis effect, improving the angiogenesis effect and increasing the inflammation regulatory effect of UC‐MSC. The safety and efficiency of UC‐MSCs therapy have been tested in clinical trials, but long‐term follow‐up for patients is needed. Although some problems and issues remain, UC‐MSCs are still a promising form of cell therapy to treat CVDs.

## CONFLICT OF INTEREST

The authors have declared that no competing interest exists.

## AUTHOR CONTRIBUTION

**Yueqiu Chen:** Conceptualization (lead); Resources (lead); Visualization (lead); Writing‐original draft (lead); Writing‐review & editing (lead). **Han Shen:** Conceptualization (equal); Investigation (equal). **Yinglong Ding:** Conceptualization (equal); Investigation (equal). **You Yu:** Formal analysis (equal); Validation (equal). **Lianbo Shao:** Validation (equal); Visualization (equal); Writing‐review & editing (supporting). **Ya Zhen Shen:** Funding acquisition (lead); Writing‐review & editing (equal).

## Data Availability

Data sharing is not applicable to this article as no new data were created or analyzed in this study.
